# Antioxidative Stress and Antiapoptosis Effect of Chitosan Nanoparticles to Protect Cardiac Cell Damage on Streptozotocin-Induced Diabetic Rat

**DOI:** 10.1155/2022/3081397

**Published:** 2022-04-25

**Authors:** Giftania Wardani, Jusak Nugraha, Mohd. Rais Mustafa, Rochmah Kurnijasanti, Sri Agus Sudjarwo

**Affiliations:** ^1^Doctoral Program of Medical Science, Faculty of Medicine, Universitas Airlangga, Surabaya, Indonesia; ^2^Study Program of Pharmacy, Hang Tuah University, Surabaya, Indonesia; ^3^Department of Clinical Pathology, Dr Soetomo Hospital, Universitas Airlangga, Surabaya, Indonesia; ^4^Department of Pharmacology, Faculty of Medicine, University of Malaya, 50603 Kuala Lumpur, Malaysia; ^5^Department of Pharmacology, Faculty of Veterinary Medicine, Univesitas Airlangga, Surabaya, Indonesia

## Abstract

The antioxidant can inhibit oxidative stress and apoptosis, which has a role in an important mechanism on diabetic-induced cardiac cell damage. The research goal was to prove the antioxidative stress and antiapoptosis effect of chitosan nanoparticles as a cardioprotector in streptozotocin-induced diabetic rats. Scanning electron microscope (SEM) and dynamic light scattering (DLS) characterize the chitosan nanoparticles. This research is a laboratory experiment which consists of the control group (rats were given distilled water), the streptozotocin group (rats were injected streptozotocin at dose of 55 mg/kg BW i.p), and the chitosan nanoparticle group (rats were given streptozotocin at dose 55 mg/kg BW i.p, and then given chitosan nanoparticles at dose 75 mg/kg BW, 150 mg/kg BW, and 300 mg/kg BW peroral). Creatine kinase-myoglobin (CK-MB) and lactate dehydrogenase (LDH) were measured from the blood sample. Malondialdehyde (MDA), superoxide dismutase (SOD), and glutathione peroxidase (GPx) from cardiac tissue were examined by ELISA; nuclear factor erythroid 2-related factor 2 (Nrf2) was evaluated by western blotting; B-cell lymphoma 2 (Bcl-2) and Caspase-3 expression were investigated by immunohistochemical staining and also were evaluated histological preparation by hematoxylin & eosin (H&E) staining. The chitosan nanoparticles have a rough surface and an irregular shape. Its size is 247.3 ± 38.1 *μ*m. Streptozotocin injection significantly increased the levels of CK-MB, LDH, MDA, and expression of caspase-3. In contrast, the levels of SOD, GPx, Nrf2, and expression of Bcl-2 decreased as compared with the control group (*p* < 0.05). This is accompanied by the loss of normal cardiac cell structure and necrosis. The administration of chitosan nanoparticles significantly reduced levels of CK-MB, LDH, MDA, and expression of Caspase-3. However, the levels of SOD, GPx, Nrf2, and expression of Bcl-2 increased as compared with the streptozotocin group (*p* < 0.05). And also, chitosan nanoparticles inhibited cell necrosis in diabetic rats. This study suggests that the administration of chitosan nanoparticles can protect cardiac cell damage in diabetic rats through antioxidative stress by decreasing ROS and increasing Nrf2 expression, level of SOD, and GPx and through antiapoptosis by increasing expression of Bcl-2 and decreasing expression of Caspase-3.

## 1. Introduction

Diabetes mellitus is a metabolic disease characterized by hyperglycemia due to insulin deficiency or resistance to insulin action or both, which can be accompanied by long-term microvascular and macrovascular complications, leading to morbidity and mortality [1, 2].

Many studies report that diabetic complications that result in cardiac cell damage are associated with oxidative stress and apoptosis. The molecular mechanism of diabetes complications seems to be multifactorial, with various consequences for cellular function. It has been reported that apoptosis occurs after oxidative stress and is enhanced during diabetes complications-induced cardiac cell damage [3–5]. Hyperglycemia in diabetes can increase advanced glycated end products (AGEs), and increased Protein Kinase C activity results in an increase in the production of superoxide in the mitochondria. This effect causes an increase in oxidative stress due to the excessive production of free radicals [6]. Prolonged hyperglycemia can lead to oxidative stress due to the overproduction of reactive oxygen species (ROS) such as O_2_^−^, OH^−^, and H_2_O_2_, which counteract cellular redox balance and cause decreased antioxidants such as SOD, GPx, and Cat. Oxidative stress causes significant damage to several cellular biomolecules, including proteins, lipids, and DNA [7–9]. The resulting dysregulated expression of many genes and proteins leads to cell apoptosis and necrosis, contributing to the progression of diabetes complications such as retinopathy, neuropathy, nephropathy, and cardiomyopathy [3, 10]. Preclinical and human research has found a relation between hyperglycemia, ROS overproduction, and the occurrence of myocyte apoptosis [11, 12].

Apoptosis has an essential role in the pathogenesis of cardiac cell damage due to complications of diabetes. Caspase-3 is one of the main executors of apoptosis, and increased caspase-3 activity indicates the presence of cell apoptosis. Bax and Bcl-2 are apoptotic and antiapoptotic proteins, with the ratio of these two proteins determining the occurrence of cell apoptosis. Bcl-2 is an antiapoptotic regulator. Bcl-2 inhibits the release of cytochrome c and the formation of the apoptosome with Apaf1, which leads to the inhibition of caspase 9 and subsequently of caspase-3 [13, 14]. New studies show that Bcl2 decreases and caspase-3 increases cardiac cell damage in streptozotocin-induced diabetic rats. Excessive ROS formation accelerates the process of apoptosis by inhibiting Bcl-2 expression and increasing caspase-3 activity, which shows that a relationship exists between oxidative stress and apoptosis [15, 16].

In addition, induction with streptozotocin in a diabetic rat model can enhance ROS and lower antioxidant enzyme effects [17, 18]. A previous study revealed that Nrf2 is a primary antioxidant regulator, and streptozotocin-induced decreased expression of Nrf2 is associated with decreased antioxidant linkage, as demonstrated by various investigators [15, 19]. Furthermore, ROS will oxidize cell membrane lipid that produces malondialdehyde (MDA) and may be used as a marker of tissue damage. The increase in MDA level indicates enhanced lipid peroxidation, causing cell necrosis or apoptosis in cardiac cell damage in diabetics. CK-MB and LDH levels in serum are used as marker enzymes of cardiac cell damage. This enzyme passes through the injured tissue and is the most effective sign of cardiomyopathy [3, 16, 20].

Several studies have shown that antioxidants can both prevent and cure cell injury induced by an increase in free radicals in the body. Exogen antioxidants like natural products have been used to avoid free radical production in streptozotocin-induced cardiac cell damage in diabetic rats [16, 21]. The crude product is used as an alternative product to antioxidants because of its cheap cost and few side effects. One of the antioxidants found in natural products is chitosan, which is used in this research to inhibit oxidative stress and apoptosis in the cardiac cell damage of diabetic rats. Researchers have extensively reported chitosan for its antioxidant activity and are utilized for studies in both in vivo and in vitro [22, 23]. This indicates that chitosan regulates the activity of antioxidant enzymes and reduces lipid peroxidation. Recently, chitosan has attracted much attention due to various biological activities associated with its antioxidant activity. Chitosan also has pharmacological properties such as hepatoprotective, nephroprotective, antiulcer, anticancer, immunostimulant, and antioxidant [24, 25]. It has been reported that chitosan is broadly used in pharmaceutical, industrial, and medical applications.

Recently, the production of natural medicine nanoparticles has played an important role in nanotechnology. The natural medicine nanoparticle has been given attention for prevention and therapeutic disease in both animals and humans. Compared to pure natural medicine, the nanoparticle-based natural treatment improved drug stability, delivery system, effectiveness, and penetration ability [26, 27]. Therefore, it is necessary to make chitosan preparations in the form of nanoparticles so the absorption, distribution, activity, and effectiveness of chitosan are improved as an antioxidant and antiapoptotic and can protect heart cell damage in diabetics.

## 2. Materials and Methods

### 2.1. Preparation of Chitosan Nanoparticles by Ball Milling Methodology

The chitosan powder (Sigma–Aldrich, Co, USA) was milled using a high-energy ball equipped with an insulating sheath and a cooling machine. The weight ratio of chitosan powder to the ball (1 : 20) in stainless steel bottles (50 ml). The container is filled to about a third of its capacity. During milling, the flask was rotated at a constant milling speed of 500 rpm for 5 h. The direction of rotation of the ball mill is changed every 30 minutes. The ball milling process is conducted at a temperature of 27°C, and the temperature is maintained with the air conditioning system to prevent overheating.

Scanning electron microscopy was used to evaluate the characteristics of the surface morphology, including the shape, size, and topography of the chitosan nanoparticles as follows: 0.1 g of chitosan nanoparticle powder was suspended in 10 ml of ethanol. Then, the suspension was sonicated for 20 minutes and dripped onto carbon tape, which stuck and dried. Furthermore, it is coated with gold and placed on a ready SEM tool so that the results of the character and size of the chitosan nanoparticles can be obtained.

And also carried out the identification particle size of chitosan nanoparticles by dynamic light scattering (DLS) (Horiba LA, Japan) as follows: to another particle, the nanoparticles must be filtered. Then, put chitosan nanoparticles in a clean cuvette until 2/3 of the cuvette is filled. After that, the cuvette containing the chitosan nanoparticle solution is inserted into the tool and closed with a sensor. Before measuring, the temperature is first set at 25°C by pressing the “Temp. Panel” menu. Standard starts counting by pressing the “Auto1” menu. Then, the tool will automatically measure the amount the particle size has been measured six times.

### 2.2. Ethical Approval

This research has been conducted through an ethical feasibility test, and all procedure experiments have been agreed upon by the Committee of the Ethical Clearance for Research in Preclinical, Faculty of Medicine, Hang Tuah University, Surabaya, Indonesia.

### 2.3. The Animal Experimental

In this experiment, we used male Wistar rats weighing about 225-250 g and aged between 2.5 and 3 months. The rat was obtained from Gajah Mada University in Yogyakarta, Indonesia. Rats were placed in a plastic cage in an air-conditioned room with a temperature maintained at 26°C. In addition, the dark and light cycles were alternated for 12 hours. The rat was given free feed, a standard commercial, and drinking water ad libitum.

### 2.4. A Diabetic Model Rat

The rat fasted overnight and then was injected with streptozotocin (Sigma Aldrich, Co, USA) at a dose of 55 mg/kg BW intraperitoneal (i.p) that was dissolved in citrate buffer (0.1 M; pH 4.5). Three days after the streptozotocin injection, blood samples were taken through the lateral vein of the tail and tested for blood glucose levels by the glucometer (Accu-Check, Roche Diagnostics, Pvt. Ltd.). Rats with a glucose level of >250 mg/dL could be used as experimental animals.

### 2.5. Experimental Designs

Forty rats were randomized into the control group (rats were given aqua dest), the streptozotocin group (rats were injected at a dose of 55 mg/kg BW i.p), and the chitosan nanoparticle group (rats were injected with streptozotocin at a dose of 55 mg/kg BW, and after 3 days, rats were given chitosan nanoparticles at doses of 75 mg, 150 mg, and 300 mg/kg BW orally once a day for 75 days). After 75 days of treatment, all the rats were anesthetized by intraperitoneal (i.p.) injection of 100 mg/kg ketamine (Ketalar, Pfizer, USA), and all groups were taken blood by intracardial to measure the level of CK-MB and LDH. The heart was collected and fixed in 10% buffered formalin for observation of heart damage in histopathological preparation. We measured the levels of MDA, SOD, and GPx by ELISA, Nrf2 expression by western blotting, expression of Bcl-2, and caspase-3 by immunohistochemical staining.

### 2.6. Measurement of MDA in Heart Tissue

MDA was measured in the supernatant of homogenized heart tissue using the thiobarbituric acid (TBA) technique, which predicts MDA production. The absorbance coefficient of the MDA-TBA complex was used to assess the level of MDA, which was measured at 532 nm. The Lipid Peroxidation Colorimetric/Fluorometric Assay Kit (BiovisionK739-100; Milpitas, CA95035, USA) was used to determine MDA levels. The results were expressed as nanomole MDA per milligram of tissue (nmol/mg tissue).

### 2.7. Measurement of Antioxidant Enzymes (SOD and GPx) in Heart Tissue

A total of 50 mg of heart tissue was washed with phosphate-buffered saline (PBS) containing 137 mM NaCl, 2.7 mM KCl, 10 mM Na_2_HPO_4_, and 1.8 mM KH_2_PO_4_ five times until clean. The sample was pounded with a mortar, then 0.5 ml of sample buffer was added, and it was centrifuged at 10,000 rpm for 10 minutes. The supernatant was taken. Rat SOD ELISA kits (Biovision-K335-100; Milpitas, CA95035, USA) were used to measure the SOD level. Similarly, GPx was also measured with the rat GPx ELISA kit t (Biovision-K762-100; Milpitas, CA95035, USA) according to the manufacturer's protocol. Enter the sample into a standard microplate incubate at 37°C for 90 minutes. Incubate the biotinylated antibody for 60 minutes at 37°C before washing three times with PBS 0.01 M. Incubate the plate at 37°C for 30 minutes with the Avidin-Biotin Complex working solution. Dish with PBS, 0.01 M. Incubates the TMB color developing agent for 30 minutes at 37°C.TMB stop solution and read the OD value on a 450 nm microplate reader. Furthermore, a standard curve is made between the value of OD and concentration so that we get the concentration SOD and GPx levels in pg/ml.

### 2.8. Immunohistochemical Staining of Bcl-2 and Caspase-3 Expression

Immunohistochemical staining was utilized to observe Bcl-2 and Caspase-3 expression. Heart tissue slices 4 *μ*m were deparaffinized and were added hydrogen peroxide at 37°C for 10 minutes to inhibit endogenous peroxide. Then, 10% normal sheep serum was given in Tris-buffered salt solution at 37°C for 30 minutes and, furthermore, incubated overnight at 4°C with anti-Bcl-2 monoclonal (1 : 100; Santa Cruz Biotechnology, USA) or anti-rat anti-caspase3 monoclonal (1 : 100, Santa Cruz Biotechnology, USA) antibodies. After that, they were washed three times with PBS and incubated with a secondary antibody from the Ultra Vision Quanto Detection System HRP DAB (Thermo Fisher Scientific, Waltham, MA, USA) for 30 minutes at room temperature and with 3.3′ diaminobenzidine (DAB) color reagent. Immunohistochemical expressions were observed by microscopy and semiquantified by Image-Pro Plus 6.0 software. The integrated optical density (IOD) of each photo was collected. Images were measured by immunoreactive area (IA) in *μ*m^2^ and IOD. The staining intensity (SI) for each image was calculated as SI = IOD/IA and the mean with a standard deviation.

### 2.9. Western Blotting Analysis of Nrf2 Protein

A total of 50 mg protein from heart tissue was subjected to electrophoresis on 12% SDS-PAGE and transferred to the nitrocellulose membrane. The membrane was blocked in 5% skim milk at room temperature for 30 minutes and then probed with rat anti Nrf2 antibody (Invitrogen, ThermoFisher, Scientific, USA) at 4°C overnight; the membranes were incubated with HRP-conjugated secondary antibody for 1 hour at 37°C. Beta-actin was used for control. Bands were visualized and analyzed by densitometry with the Typhoon FLA 9500 (GE Healthcare, Upsala, Sweden).

### 2.10. Measurement of CKMB and LDH

The enzymes CK-MB and LDH, which are linked to heart cell damage, were tested in the serum. The experiments were carried out using commercially available test kits (Sigma-Aldrich Corp., MO, USA) and following the manufacturer's instructions.

### 2.11. Histopathological Examination

Rat's heart tissue was collected and fixed in 10% buffered formalin solution, dehydrated in ethanol, and embedded in paraffin. Heart tissue sectioned at 4 *μ*m was stained with hematoxylin and eosin. The photomicrographs of each tissue section were observed using cell imaging software for life science microscopy (Olympus Soft Imaging Solution GmbH, Munster, Germany).

### 2.12. Statistical Analysis

The data were presented in the form of mean ± standard deviation. The one-way analysis of variance (ANOVA) is used to analyze the data and will be continued with the LSD test through the application of SPSS 17.0 (SPSS Inc, Chicago, USA).

## 3. Results

### 3.1. Characterization of Chitosan Nanoparticles

**Figure 1:**
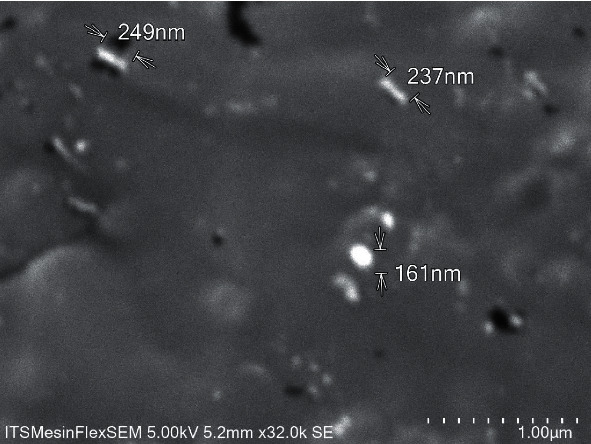
Chitosan nanoparticles in scanning electron microscope.

Chitosan nanoparticle in scanning electron microscopy (SEM) shows a rough surface morphology and an uneven shape (Figure 1).

**Figure 2:**
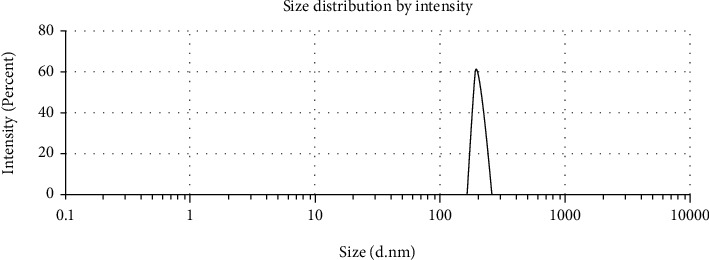
Size distribution of chitosan nanoparticles by dynamic light scattering.

DLS shows that the chitosan nanoparticles particle size is approximately 247.3 ± 38.1 *μ*m (Figure 2).

### 3.2. The Effect of Chitosan Nanoparticles on MDA, SOD, and GPx of Diabetic Rat Heart

The results of the ELISA assay demonstrated that the streptozotocin-induced cardiac cell damage significantly increased in the level of MDA and decreased in the level of SOD and GPx as compared with the control group (*p* < 0.05). However, we found that dose-dependent the administration of chitosan nanoparticles and only at dose 300 mg/kg BW significantly reduced the level of MDA and enhanced the level of SOD and GPx as compared with the streptozotocin group (*p* < 0.05) (Table 1).

### 3.3. The Effect of Chitosan Nanoparticles on Nrf2 Protein Expression of Diabetic Rat Heart

**Figure 3:**
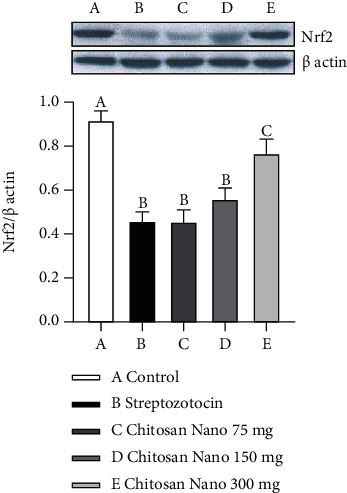
Western blot analysis of Nrf 2 protein expression of rat heart tissue. Nrf2 expression in rat heart control group (a); streptozotocin group (b); and chitosan nanoparticle group at dose 75 mg/kg BW (c), 150 mg/kg BW (d), and 300 mg/kg BW (e). ^a-c^Columns that have different letters statistically differ (*p* < 0.05).

Western blot analysis revealed that Nrf2 protein expression was significantly decreased in heart of the streptozotocin-induced diabetic rat (*p* < 0.05) as compared with the control group (Figure 3). However, dose-dependent administration of chitosan nanoparticles and only at dose 300 mg/kg BW significantly increased Nrf2 protein expression as compared with streptozotocin-induced diabetic rats.

### 3.4. Effect of Chitosan Nanoparticle on Bcl-2 of Diabetic Rat Heart

**Figure 4:**
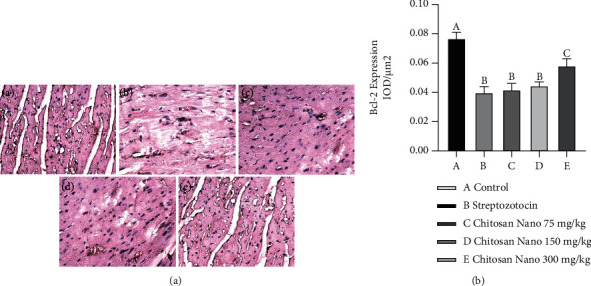
Photomicrographs of immunohistochemical staining of Bcl-2 expression of rat heart tissue. (4A) Bcl-2 expression in rat heart (black arrow) from the control group (a); streptozotocin group (b); and chitosan nanoparticles group at a dose 75 mg/kg BW (c), 150 mg/kg BW (d), and 300 mg/kg BW; (4B) IOD/*μ*m^2^ shows Bcl-2 expression from semiquantitative evaluation (400×).

To prove that chitosan nanoparticles are involved in apoptosis on streptozotocin-induced diabetic rats, we measured antiapoptosis Bcl-2 expression by immunohistochemical staining. The administration of streptozotocin significantly decrease Bcl-2 expression compared to the control group (*p* < 0.05). However, dose-dependent administration of chitosan nanoparticles could increase the expression of Bcl2 (Figure 4).

### 3.5. Effect of Chitosan Nanoparticle on Caspase-3 of Diabetic Rat Heart

**Figure 5:**
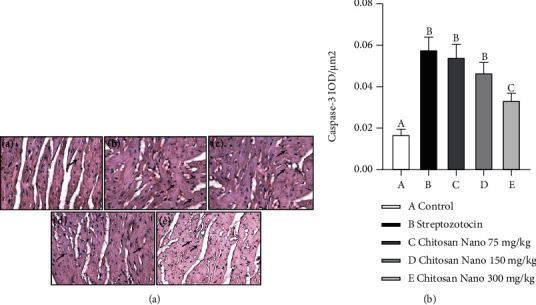
Photomicrograph of immunohistochemical staining of Caspase-3 expression of rat heart tissue. (A) Caspase-3 expression in rat heart (black arrow) from the control group (a); streptozotocin group (b); and chitosan nanoparticles group at dose 75 mg/kg Bw (c), 150 mg kg/BW (d), and 300 mg/kg BW. B. IOD/*μ*m^2^ shows Caspase-3 expression from semiquantitative evaluation (400×).

To prove that chitosan nanoparticles are involved in apoptosis on streptozotocin-induced diabetic rats, we measured executioner Caspase-3 expression by immunohistochemical staining. The administration of streptozotocin significantly increase Caspase-3 expression compared to the control group (*p* < 0.05). However, dose-dependent administration of chitosan nanoparticles could decrease caspase-3 (Figure 5) (*p* < 0.05).

### 3.6. The Effect of Nanoparticle Chitosan on the Serum Level of CK-MB and LDH of Diabetic Rat

As a marker of cardiac cell injury, the serum level of CK-MB and LDH was significantly increased in the streptozotocin-induced diabetic rat (*p* < 0.05). Following administration of chitosan nanoparticles only at a dose of 300 mg/kg BW significantly decreased level of the CK-MB and LDH (*p* < 0.05) (Table 2).

### 3.7. The Effect of Chitosan Nanoparticles on the Structural Change of Diabetic Rat Heart

**Figure 6:**
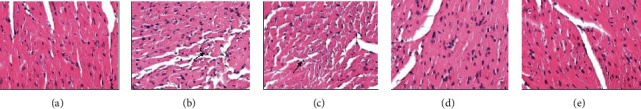
Histological study of administration of chitosan nanoparticle on streptozotocin-induced cardiac cell damage. The controls group showed normal morphology of the heart (a). The treatment streptozotocin group showed necrosis (indicated by black arrows) (b). Administration of chitosan nanoparticle 75 mg/kg BW and 150 mg/kg BW showed necrotic changes (c and d). However, administration of chitosan nanoparticle 300 mg/kg showed regeneration on cardiac cells damage (e) H and E, ×400.

To evaluate the nephroprotective effect of chitosan nanoparticles, we conducted a histopathological examination of streptozotocin-induced cardiac cell injury in diabetic rats. Light microscope investigation revealed that the control group is the normal structure of the heart. The administration of streptozotocin can cause morphological irregularities, several cardiac cell degeneration, and necrosis (Figure 6). The treatment with chitosan nanoparticles could inhibit cardiac cell necrosis and protect the normal structure of the heart.

## 4. Discussion

Many researchers have developed an interest in synthesizing nanoparticles to enhance their biological activity, especially those used as antioxidant agents. A natural product of nanotechnology can increase bioavailability, biodistribution, sensitivity, and reduce pharmacological toxicity [26, 27]. The milling process is conducted to make chitosan nanoparticles. This research shows that the particle size of chitosan nanoparticles is 247.3 ± 38.1 *μ*m, which is expected to increase the effectiveness of antioxidative stress and antiapoptosis.

Oxidative stress and apoptosis often coexist in hyperglycemia and have been reported to have a role in multiorgan complications in diabetes mellitus. Overproduction of ROS leads to oxidative stress due to increased glucose autooxidation in hyperglycemia. Furthermore, ROS can trigger apoptosis signals, leading to cell damage [3, 4, 6, 11].

Streptozotocin-induced hyperglycemia in rats has been described as a useful experimental diabetes model for studying diabetic complications. Streptozotocin can interfere with the function of the beta cells of the islets of Langerhans, resulting in inhibition of insulin release, which in turn leads to hyperglycemia and diabetic complications like diabetic cardiomyopathy [6, 16]. The aim of the study was to evaluate the potency of antioxidative stress and antiapoptosis of chitosan nanoparticles to protect against streptozotocin-induced diabetic cardiomyopathy in rats.

The present study has revealed that when compared to the control group of rats, those injected with streptozotocin significantly exhibited elevated malondialdehyde and reduced endogenous antioxidant enzymes the SOD and GPx levels in cardiac tissue. Several investigators reported that the MDA level marker of lipid peroxidation was increased in hyperglycemia rats due to increased free radical production. Streptozotocin increases the intensity of oxidative stress through autooxidation of monosaccharides leading to excessive production of superoxide and hydroxyl radicals, which can decrease the level of SOD and GPx. These radical species can oxidize polyunsaturated fatty acid membrane lipids to increase malondialdehyde, which results in necrosis in cardiac tissue [7, 20]. Various researchers have shown that streptozotocin can decrease the expression of the antioxidant responsive protein, Nrf2, thereby reducing the formation of antioxidants such as SOD and GPx [6, 15, 21]. In the diabetic rat, the oral administration of chitosan nanoparticles only at a dose of 300 mg/kg BW decreases MDA level and increases SOD and GPx levels significantly compared with the streptozotocin-induced diabetic cardiomyopathy. In recent years, chitosan has attracted much attention because of various biological activities related to its antioxidant activity and are utilized for studies in both in vivo and in vitro. This finding is supported by previous researchers who stated that chitosan has extreme antioxidant activity and can scavenge free radicals. The antioxidant activity of chitosan as a free radical scavenger can be due to the ^−^OH group in the polysaccharide unit of chitosan that can react with hydroxyl free radicals (∗OH) in the test of the hydroxyl radical assay. Also, the -NH_2_ groups of the chitosan can form ammonium groups (NH^3+^) by absorbing H^+^ ions and reacting with the ^−^OH radical through addition reaction to form stable macromolecules. As the intramolecular and intermolecular hydrogen bonds in chitosan molecules are solid (high bond dissociation energy), the ^−^OH and -NH_2_ groups are difficult to separate and react with hydroxyl free radicals, in agreement with previous reports that the scavenging of ROS by chitosan may in part be related to increased activities of antioxidant enzymes and decreased levels of MDA. The cells possess an intricate network of defense mechanisms, including antioxidant compounds such as SOD and GPx, to neutralize excessive ROS accumulation [24, 27, 28]. In addition, Nrf2 is considered a master regulator of the antioxidant response, and various researchers have shown increased expression of Nrf2 by chitosan. The reason for this enhanced Nrf2 expression in the cardiac cells could be the chitosan-caused decreased intracellular ROS, which can increase antioxidant [28].

On the other hand, antiapoptosis has also been associated with cardiomyopathy in diabetic patients. This study showed that induction with streptozotocin could reduce the expression of Bcl-2, whereas the expression of caspase-3 increased in the heart of rats. Several researchers have proven that overproduction of ROS in oxidative stress could decrease expression of the antiapoptosis protein, Bcl-2, and increase expression of caspase-3, which lead to the fragmentation of DNA and apoptosis in the heart of rats after streptozotocin administration [6, 7]. This could be due to the reduced expression of Nrf2 by streptozotocin. Nrf2 is considered a master regulator of the antioxidant response, and decreased expression of Nrf2 by streptozotocin has been shown by various researchers [19]. However, dose-dependent oral administration of chitosan nanoparticles could increase the expression of Bcl-2 and decrease the expression of caspase-3 in the heart of diabetic rats. This study demonstrated that chitosan nanoparticles with free radical scavenging ability to decrease ROS generation and enhance endogenous antioxidant activity such as SOD and GPx, leading to decreased oxidative stress and apoptosis that play an important role in the pathogenesis of various diabetes complications such as cardiomyopathy [29, 30]. The scavenging mechanism of chitosan is related to their hydrogen donating ability to free radicals to form stable molecules. Here, we clearly show that administration of chitosan nanoparticles on diabetic rats can reduce ROS formation and increase the level of antioxidant enzyme (SOD and GPx), resulting in an increase in Bcl-2 expression and a decrease caspase-3 expression, which has a vital role in cardiac cell apoptosis. Animal and human studies have found a correlation between hyperglycemia, ROS overproduction, and frequency of cardiac cell apoptosis. ROS inhibition with an antioxidant pretreatment reduced the intensity of oxidative stress and cardiac cell apoptosis [4, 6]. Recent studies have demonstrated that chitosan induced nuclear factor erythroid-derived2-like2 (Nrf2) activation. Nrf2, encoded by the gene NFE2L2, is a master regulator of phase II antioxidant enzyme that plays an essential role in the cellular protection against free radical damage and reduces the incidence of extremely derived degenerative diseases such as diabetic complications [31]. This result suggested that chitosan nanoparticles on diabetic rats can increase the level of antioxidant enzyme (SOD and GPx), resulting in an increase in Bcl-2 expression and a decrease in caspase-3 expression through Nrf2 activation.

In the results, we also showed that functional heart impairment in streptozotocin-induced diabetic cardiomyopathy was evidenced by the increased levels of biochemical markers such as CK-MB and LDH, which were significantly higher than the control group. The administration of chitosan nanoparticles at a dose of300 mg/kg BW but not at a dose of 75 mg/kg BW and 150 mg/kg BW significantly reduced CK-MB and LDH levels in the heart cell damage of diabetic rats. Streptozotocin-induced cardiomyopathy is characterized by elevated serum CK-MB and LDH levels that are associated with increased ROS formation in oxidative stress. The ability of antioxidants to remove ROS has been demonstrated to play an important role in contributing to cardioprotective efficacy [5, 16]. The administration of chitosan nanoparticles significantly inhibits streptozotocin-induced heart cell damage, which is related to reduced CK-MB and LDH levels, suggesting that scavenger free radical of chitosan nanoparticles has the effect ability to inhibit heart cell damage caused by streptozotocin. These results agree with previous research that the antioxidant enzymes can interfere with streptozotocin-induced cardiomyopathy, as shown by decreased CK-MB and LDH levels. The reduced release of CK-MB and LDH in the treatment groups demonstrated the protective effects of chitosan nanoparticles through antioxidants [24, 25].

In the present research, the histological investigation showed that the administration of streptozotocin caused necrosis in the cardiac cells. Recently, has been the realization that ROS-mediated peroxidation of membrane lipids and ROS-induced fragmentation of DNA is associated with necrosis and apoptosis [6, 17]. Therefore, inhibition of oxidative damage by supplementation of antioxidants becomes an attractive therapeutic strategy to reduce the risk of diabetic complications such as cardiomyopathy. The administration of the chitosan nanoparticles can protect against streptozotocin-induced heart damage. The histological observation clearly indicates that the administration of chitosan nanoparticles remarkably decreased necrosis in the cardiac cells induced by streptozotocin. This study shows that chitosan nanoparticles have cardioprotective activity through antioxidants and antiapoptosis. Therefore, it is hoped that chitosan nanoparticles can be used to help people with diabetes to prevent complications such as retinopathy, nephropathy, atherosclerosis, and cardiomyopathy.

## 5. Conclusion

In conclusion, this study suggests that the administration of chitosan nanoparticles can protect cardiac cell damage in diabetic rats through antioxidative stress by decreasing ROS and increasing Nrf2 expression, level of SOD, and GPx and through antiapoptosis by increasing expression of Bcl-2 and decreasing expression of Caspase-3.

## Figures and Tables

**Table 1:** Effects of chitosan nanoparticle on in the level of MDA, SOD, and GPx of diabetic rat heart.

Group	Means ± standard deviation		
	MDA (nmol/mg tissue)	SOD (U/mg tissue)	GPx (U/mg tissue)
Control group	58.9^a^ ± 4.2	11.3^a^ ± 1.01	1.56^a^ ± 0.10
Streptozotocin group	82.4^b^ ± 6.4	7.17^b^ ± 0.83	0.94^b^ ± 0.09
Chitosan nano 75 mg/kg BW	78.5^b^ ± 4.3	8.42^b^ ± 1.13	0.87^b^ ± 0.13
Chitosan nano 150 mg/kg BW	72.8^b^ ± 4.6	5.12^b^ ± 0.81	0.91^b^ ± 0.15
Chitosan nano 300 mg/kg BW	64.6^c^ ± 3.7	7.17^c^ ± 1.07	1.09^c^ ± 0.09

^a,b,c^Different superscript within each column indicate a significant difference between the mean (*p* < 0.05).

**Table 2:** The effect of chitosan nanoparticle on the serum level of CK-MB and LDH of diabetic rat.

Group	Means ± standard deviation	
	CK-MB (IU/L)	LDH (IU/L)
Control Group	70.4^a^ ± 6.8	101.4^a^ ± 8.4
Streptozotocin group	106.7^b^ ± 8.6	152.6^b^ ± 13.6
Chitosan nano 75 mg/kg BW	101.4^b^ ± 6.9	147.3^b^ ± 10.8
Chitosan nano 150 mg/kg BW	93.8^b^ ± 7.1	139.7^b^ ± 9.9
Chitosan nano 300 mg/kg BW	79.5^c^ ± 5.7	124.5^c^ ± 8.7

^a,b,c^Different superscript within each column indicate a significant difference between the means (*p* < 0.05).

## Data Availability

The data used to support the findings of this research are included within the article.
